# Characterization of a thermostable uricase derived from *Thermoactinospora rubra* YIM 77501^T^ and its heat-resistant mechanism

**DOI:** 10.3389/fmicb.2025.1615845

**Published:** 2025-06-19

**Authors:** Xin Yan, Lan-Xin Tang, Maite Ortúzar, Li-Quan Yang, Peng Sang, Na-Ying Jin, Lin-Hua Li, Zheng-Feng Yang, Yan-Wen Wang, Wen-Jun Li, Wei Hu, Yi-Rui Yin

**Affiliations:** ^1^College of Agriculture and Biological Science, Dali University, Dali, China; ^2^Xizang Key Laboratory of Plateau Fungi, Institute of Plateau Biology of Xizang Autonomous Region, Lhasa, China; ^3^Department of Cardiology, The First Affiliated Hospital of Kunming Medical University, Kunming, China; ^4^State Key Laboratory of Biocontrol, Guangdong Provincial Key Laboratory of Plant Resources and Southern Marine Science and Engineering Guangdong Laboratory (Zhuhai), School of Life Sciences, Sun Yat-sen University, Guangzhou, China; ^5^Key Laboratory of Bioinformatics and Computational Biology, Department of Education of Yunnan Province, Dali University, Dali, China; ^6^Cangshan Forest Ecosystem Observation and Research Station of Yunnan Province, Dali University, Dali, China; ^7^Co-Innovation Center for Cangshan Mountain and Erhai Lake Integrated Protection and Green Development of Yunnan Province, Dali University, Dali, China

**Keywords:** uricase, *Thermoactinospora rubra* YIM 77501^T^, thermostable, heat-resistant mechanism, degrade uric acid

## Abstract

**Introduction:**

Uricases are oxidative enzymes that catalyze the conversion of uric acid to allantoin and hydrogen peroxide, widely utilized in uric acid testing and the treatment of gout, hyperuricemia, and Tumor Lysis Syndrome (TLS). The search for uricases with long-term thermostability has become a significant area of research.

**Methods:**

In this study, a uricase gene (truox) was obtained from the genome of *Thermoactinospora rubra* YIM 77501^T^, which was subsequently cloned and heterologously expressed. The resulting enzyme, TrUox, was comprehensively characterized for its biochemical properties and analyzed through molecular dynamics (MD) simulations.

**Results:**

TrUox exhibits maximal catalytic activity at 35°C and pH 7.6 (mesophilic range). Notably, its thermostability is exceptional: the enzyme retains over 90% residual activity after 4 days of incubation at 50°C (with activity measured post-thermal treatment at 35°C) and maintains >90% activity for 10 days at physiological temperature (37°C). *In vitro*, 1.14 μg/mL TrUox effectively lowered serum uric acid (UA) from >700 to < 420 μM within 2 h in hyperuricemic models. MD simulations comparing TrUox with Rasburicase indicate it's more rigid/stable globally, less flexible, has fewer sub-states, and is more stable in FEL.

**Discussion:**

These results demonstrate TrUox as a robust uricase exhibiting dual advantages of catalytic efficiency and enhanced thermostability, positioning it as a promising biocatalyst for industrial-scale production and therapeutic development. Our preliminary study into its thermostable mechanism provides a theoretical foundation for future production and research.

## Introduction

Uric acid, a byproduct of purine metabolism, is converted to the more soluble compound allantoin by the enzyme uricase or urate oxidase (EC 1.7.3.3), facilitating its excretion in urine (Wu et al., 1989). However, evolutionary mutations in the uricase gene have resulted in the loss of this enzyme's activity in humans and other primates, thereby establishing uric acid as the end product of purine metabolism in humans (Oda et al., 2002; Maiuolo et al., 2016). This evolutionary change predisposes humans to higher uric acid levels compared to other mammals, thereby increasing the risk of hyperuricemia-related diseases (Du et al., 2024; Wen et al., 2024). Modern dietary patterns and lifestyle changes have led to increased purine consumption, further elevating blood uric acid levels, which can crystallize and cause comorbidities hyperuricemia and gout (Singh and Gaffo, 2020; Timsans et al., 2024; De Becker et al., 2019). These conditions demonstrate significant associations with comorbidities including hypertension, hyperlipidemia, cardiovascular disease, obstructive sleep apnea, and chronic kidney disease (Hansildaar et al., 2021; McCormick et al., 2020; Felten et al., 2020).

Clinically, pharmacological strategies for modulating and reducing uric acid levels primarily encompass three categories: drugs inhibiting uric acid synthesis, uricosuric agents that promote its excretion, and urate oxidase preparations (Terkeltaub, 2023). While these first two drug classes can control uric acid levels to a certain extent, their therapeutic efficacy is often limited by inter-individual variability and they are associated with specific adverse effect profiles; furthermore, they do not exert a direct or rapid lytic effect on pre-existing urate crystal deposits (Hotea et al., 2023). In contrast, urate oxidase efficiently catalyzes the enzymatic conversion of uric acid to the more soluble allantoin, thereby rapidly lowering serum uric acid concentrations and significantly promoting the dissolution of established urate crystal deposits (Zhang et al., 2022). However, the widespread clinical application of exogenous urate oxidase remains constrained by factors such as its inherent high immunogenicity and relatively short biological half-life (Cho et al., 2025). Taking the clinically commonly used recombinant uricase, Elitek (rasburicase), as an example, its reported half-life is 17–21 h; although a single administration can rapidly reduce serum uric acid levels by 80%−90%, the duration of this efficacy is only about 24 h (Sanofi-Aventis U.S. LLC, 2023). The loss of uricase activity is primarily due to the disruption of its tertiary and secondary structures (Caves et al., 2013). To enhance the stability of urate oxidase and optimize its therapeutic potential, various strategies have been explored, such as amino acid mutations, disulfide bond introduction (Akhlaghi et al., 2025; Zhu et al., 2022), PASylation (genetic fusion of a polypeptide composed of Proline, Alanine, and/or Serine repeats, PAS, to the N- or C-terminus of target proteins (Mirzaeinia et al., 2020), and enhancing inter-subunit hydrogen bonding with organic reagents (Liu et al., 2009). Despite these effort, there remains a lack of highly thermostable uricases for hyperuricemia treatment.

*Thermoactinospora rubra* YIM 77501^T^, a strictly aerobic, Gram-positive, thermophilic actinobacterium isolated from volcanic sandy soil, demonstrates both sporulation properties and cellulose-degrading capabilities (Yin et al., 2017a,b). These traits suggest its potential to harbor thermoadaptive enzyme systems. Enzymes derived from such thermophilic microorganisms typically demonstrate remarkable thermostability and extreme pH stability, traits closely linked to the selective pressures of their native environment (Haki, 2003). The search for thermostable enzymes often focuses on microorganisms from thermal environments, as thermotolerant bacteria processes unique genetic, physiological, and regulatory mechanisms that confer enzyme stability (Wang et al., 2015; Atalah et al., 2019). These enzymes typically exhibit increased hydrophobicity, ionic bonding, lower ring lengths and flexibility, and stronger N-and C-terminal interactions compared to mesophilic enzymes. Despite phylogenetic distance between heat-tolerant and expressing bacteria, the thermal properties of these enzymes are retained when cloned and expressed in mesophilic hosts (Vieille and Zeikus, 2001).

In this study, we identified, cloned, and characterized a uricase gene from the thermophilic actinomycete *T. rubra* YIM 77501^T^ (Zhou et al., 2012). The heterologously expressed uricase was analyzed for thermostability and compared to Rasburicase (AfUox), a commonly used mesophilic enzyme derived from *Aspergillus flavus* (Collings et al., 2010), using molecular dynamics simulations. This comparison revealed key differences in global conformational stability and free energy landscapes, providing valuable insights into enhancing uricase thermostability for pharmaceutical and clinical applications.

## Materials and methods

### Strain and culture media

*Thermoactinospora rubra* YIM 77501^T^ (=DSM 45614^T^= CCTCC AA 2011014^T^) was cultured on modified ISP 2 medium at 50°C (Zhou et al., 2012) for DNA extraction. *Escherichia coli* DH5α was utilized for gene cloning and expression. Recombinant E. coli was cultured in Luria-Bertani (LB) medium containing 50 μg/mL kanamycin at 37°C.

### Construction and transformation of recombinant plasmid

Genomic DNA from *T. rubra* YIM 77501^T^ was extracted using the DNA Purification kit (Sangon Biotech, China) according to the manufacturer's instructions. Primers were designed based on the sequence in the database: 77501-Uox-F (CATCATCATCATCATCATGAAATGTCAGTCATCCTTGGCCCGAA) and 77501-Uox-R (GTGCTCGAGTGCGGCCGCAAGCTCCCAGGCGAAGGCCGCGTCG). The uricase gene (truox, NCBI accession NO.: WP_084956803) was amplified using TransStarFastPfu Fly DNA Polymerase (TransGen Biotech, China). PCR conditions included initial denaturation at 98°C for 3 min, followed by 30 cycles at 98°C for 20 s, 55°C for 30 s, and 72°C for 1 min, and a final extension at 72°C for 5 min for the final extension. PCR products were analyzed on a 1.2% agarose gel, and inserted into the pSHY211 plasmid (Yin et al., 2023) using the pEASY-Uni Seamless Cloning and Assembly Kit (TransGen Biotech, China) to construct pSHY211-truox.

#### Expression and purification

Recombinant *E. coli* DH5α with pSHY211-truox was cultured in LB medium at 37°C with shaking at 180 rpm for 12 h. Cells were collected by centrifugation at 12,000 × g at 4°C for 15 min resuspended in PBS (10 mM imidazole, pH 7.6), and lysed ultrasonically. After centrifugation, cell-free extracts were purified using the Ni-chelating affinity column (Histrap, TransGen Biotech, China). The column was washed sequentially with 50 mL PBS containing 10, 20, and 30 mM imidazole, followed by gradient elution with 10 mL PBS containing 50, 100, 150, and 200 mM imidazole. The purified proteins were desalted using D-10 Desalting Columns (GE, USA) and analyzed by 12% SDS-PAGE. Protein concentration was determined by the Bradford method with Bradford Protein Assay Kit (Order NO. C503031, Sangon Biotech, China) using bovine serum albumin as the standard.

### Enzyme assays

Uricase activity was assayed in a 1.0 mL reaction system containing a working solution of uric acid (0.04 mol/L), EDTA (1 mmol/L), boric acid (50 mmol/L), and Triton X-100 (0.001%) at pH 8.5. The reaction was terminated by adding 20% KOH. Enzyme activity was quantified by monitoring the decrease in absorbance at 290 nm after 30 min incubation at 35°C and pH 7.6. One unit of enzyme activity was defined as the amount of enzyme required to convert 1 μmol of uric acid to allantoin per minute under these conditions.

The thermostability of TrUox was evaluated by incubating the enzyme at different temperatures (4, 35, 37, 50°C) and measuring residual activity at 2-day intervals over a 18-day period, with the initial time point (0-day) serving as the untreated control. pH stability was evaluated in buffers ranging from pH 4.0 to 10.0 at 4°C for 12 and 24 h, with the 0-h time point serving as the untreated control. Metal ion and chemical reagent tolerance were tested with 1 mmol/L of different metal ions and 1% chemical reagents, with reactions without additives as control. The *K*m and Vmax values of urate oxidase were calculated using the Lineweaver-Burk method, based on the reduction of uric acid absorbance at 290 nm over a 10-min period.

Guidelines from ACR and EULAR, along with others like ASCR (American Society of Clinical Rheumatologists), 3Es (Evidence, Expertise, Exchange), JSGNM (Japanese Society of Gout and Nucleic Acid Metabolism), and DCGP (Dutch College of General Practitioners) define normal blood uric acid levels as below 6 mg/dL (360 μmol/L; Ruoff and Edwards, 2016). Furthermore, most studies indicate that males have higher uric acid levels than females, particularly before menopause. Hence, the normal range for men is 3.4–7.0 mg/dL (~202–416 μmol/L), while for women it is 2.4–6.0 mg/dL (~143–357 μmol/L). Consequently, this study adopted 360–420 μmol/L as the standard normal uric acid range (Singh and Gaffo, 2020). The enzyme solution was reacted with a 600 μmol/L uric acid stock solution in saline buffer at 37°C pH 7.4. Uric acid content was measured at regular intervals until the OD_290_ value stabilized, matching the baseline (0 μmol/L of uric acid), at which point the reaction was terminated. For the degradation experiment of TrUox in saline solution, the minimum enzyme dose was added to anticoagulated blood samples from six hyperuricemic volunteers, whose blood uric acid levels were 715, 731, 618, 487, 717, and 757 μM, respectively. For each anticoagulated blood sample collected from the hyperuricemic volunteers, triplicate technical replicates were performed by independently processing three aliquots (300 μL each) derived from the same 1 mL specimen. Measurements were conducted under identical experimental conditions to assess analytical consistency. The uric acid concentration in the blood was continuously monitored every 1 h using a uric acid detector (Kefu Yi uric acid tester UA03-C, China).

### Homology modeling and quality evaluation

The present study employed the SWISS-MODEL template library to perform homology modeling analysis of the TrUox protein. The results revealed that it shares the highest sequence identity (53.85%) with the X-ray crystal structure template 7F2V (PDB ID), while demonstrating complete conservation of catalytic residues. The quality, stability, and accuracy of the TrUox structure were modeled and evaluated using VERIFY 3D and ProQ tools (Chiu et al., 2021b).

### MD simulations

The initial structure of AfUox for molecular dynamics (MD) simulations was retrieved from the Protein Data Bank (PDB: 4D12). Simulations were performed using the GROMACS-2021.5 software with the following parameters and protocols: the AMBER99SB-ILDN force field (Pires et al., 2011; Abraham et al., 2015) and TIP3P water model (Jorgensen et al., 1983) were applied. Both structural models were centered in a dodecahedral box with a minimum distance of 1.0 nm between the protein and box edges. To neutralize the system and mimic physiological salt conditions, the box was solvated with water molecules, and appropriate amounts of Na^+^ and Cl^−^ ions were added by replacing equivalent volumes of water. Energy minimization was conducted using the steepest descent algorithm to resolve atomic clashes and stereochemical conflicts.

Prior to the production phase of MD simulations, the system underwent restrained equilibration under NVT (constant number of atoms, volume, and temperature) and NPT (constant number of atoms, pressure, and temperature) ensembles. Production MD simulation parameters were set as follows: an integration time step of 2 fs; covalent bond constraints implemented via the LINCS algorithm (Hess et al., 1997); long-range electrostatic interactions treated using the Particle Mesh Ewald (PME) method (Darden et al., 1993) with a cutoff radius of 1.0 nm; van der Waals (vdW) interactions modeled by the Lennard-Jones potential with a cutoff radius of 1.0 nm. The system temperature was maintained at 300 K using the v-rescale thermostat (Bussi et al., 2007) with a coupling time constant (τ_t) of 0.1 ps, while pressure was controlled at 1 atm via the Parrinello-Rahman barostat (Nosé and Klein, 1983; Parrinello and Rahman, 1981) with a coupling time constant (τ_p) of 0.5 ps. To enhance conformational sampling efficiency, a multi-replica strategy was employed, with three independent 200 ns production MD simulations conducted for each system, each initiated with distinct atomic velocities assigned by Maxwell-Boltzmann distributions at 310 K. Root-mean-square deviation (RMSD) and Cα root-mean-square fluctuation (RMSF) were calculated using the GROMACS tools “gmx rms” and “gmx rmsf”, respectively (Chiu et al., 2021b; Sang et al., 2016).

### Free energy calculations

The free energy landscape (FEL) of TrUox and AfUox was constructed based on projections on eigenvectors PC1 and PC2 from the simulation trajectories, using the probability density function:


$$\begin{array}{l} F\left(s\right)=-k_{\text{B}}T\text{ ln}\left(N_{i}/N_{max}\right) \end{array}$$

Where, k_B_ is Boltzmann constant, T is the temperature, N_i_ is the probability that the protein conformation is at I, and N_max_ is the maximum probability (free energy of state i).

### Statistical analysis

Unless otherwise stated, all experiments were performed in triplicate, and the mean values were used for all analyses. Data were analyzed using SPSS 20.0 statistical software and are expressed as mean ± standard deviation (SD). Statistical comparisons between multiple treatment groups were performed using one-way ANOVA followed by Tukey's test. A *p*-value < 0.05 was considered statistically significant in all comparisons.

## Results and discussion

### Amplification, expression, and purification of TrUox

The open reading frame (ORF) of the uricase gene from *T. rubra* YIM 77501^T^ was amplified and analyzed using agarose gel electrophoresis, revealing a band ~900 bp closely corresponding to the sequencing result of 867 bp. The uricase, TrUox, was subsequently examined for its translation and physicochemical properties using the ExPASy tool (https://www.expasy.org/), predicting a composition of 288 amino acids and an isoelectric point of 5.75, classifying it as a stable acidic protein. A comparative analysis of the TrUox amino acid sequence with related urate oxidase in the NCBI database revealed a 84.72% sequence similarity to urate oxidase from *Nonomuraea soli* with a phylogenetic constructed using MEGA 7 software package (Supplementary Figure S1).

**Figure 1:**
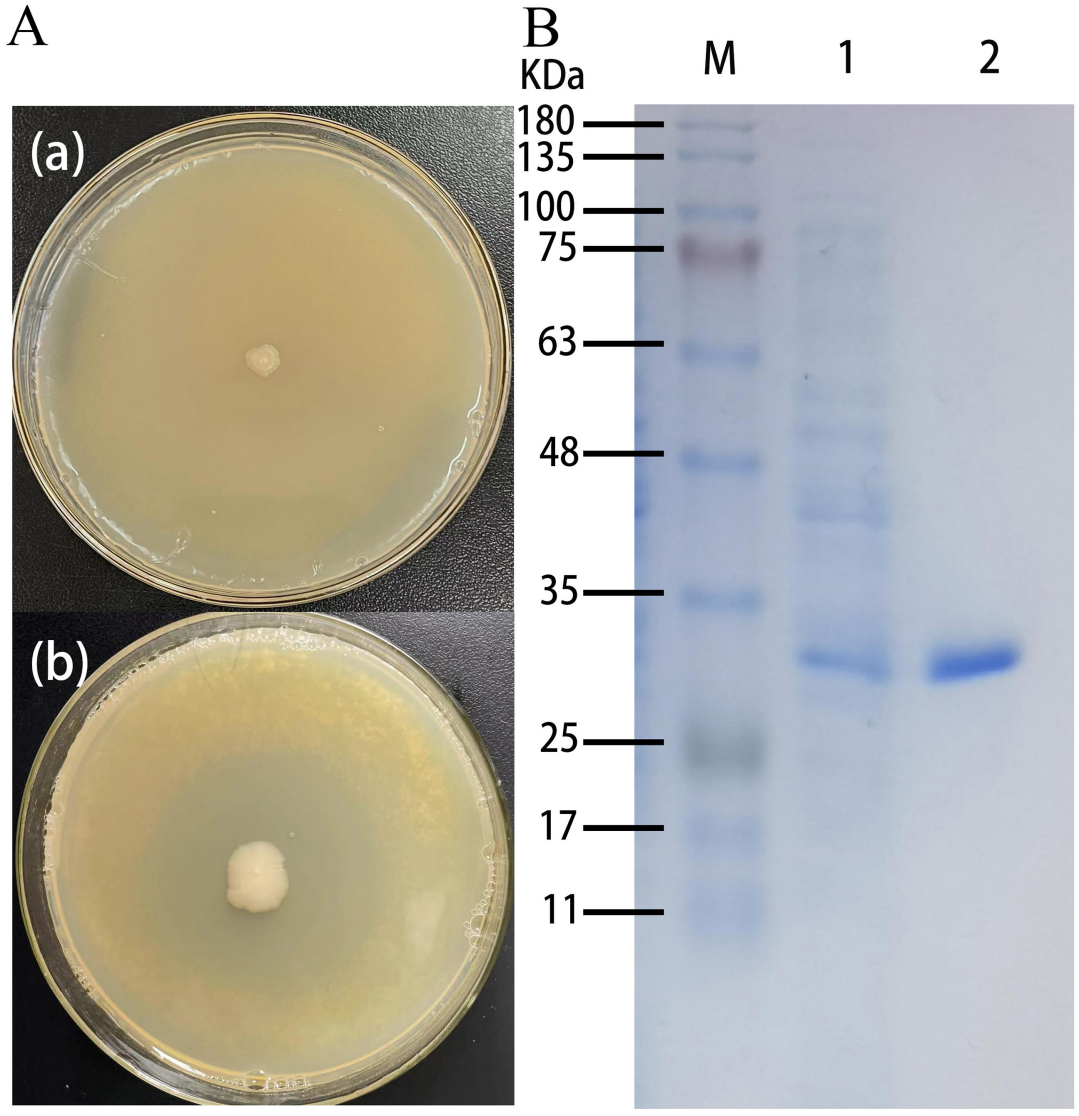
Control plate and positive recombinant pSHY-TrUox strain displaying a degradation halo of uric acid **(a)**. SDS-PAGE of uricase expression in *Escherichia coli*, a: *Escherichia coli* DH5α, b: pSHY-TrUox, M: Marker, 1: crude enzyme solution of cell disruption, 2: purified TrUox **(b)**.

Positive recombinant pSHY-TrUox strains were screened on LB agar plates supplemented with 2 g/L uric acid, as evidenced by clear zones of uric acid degradation around the colonies (Figure 1a). This result confirms the successful cloning of pSHY-TrUox and its functional expression. Enzyme viability assays were conducted using samples from both the fermentation broth and cell lysates during the fermentation process. The assays established that the uricase expressed by pSHY-TrUox functions as an intracellular enzyme. SDS-PAGE analysis of the purified TrUox displayed a distinct protein band at 30 kDa (Figure 1b), consistent with the expected molecular weight of 31.871 kDa.

### Identification of enzyme properties

**Figure 2:**
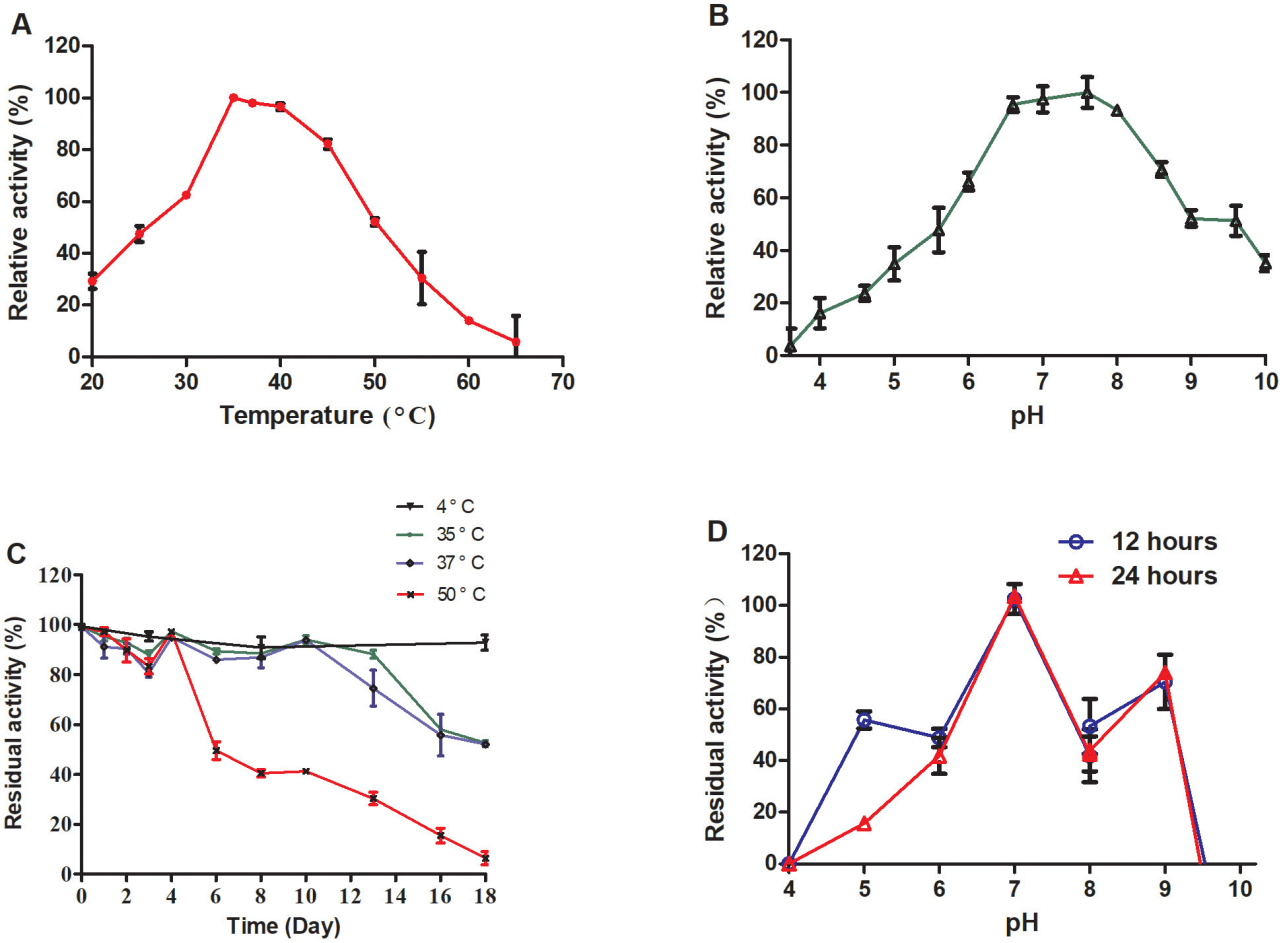
Effects of temperature and pH on enzyme activity and stability. The relative enzyme activity curve of TrUox was measured at varying temperatures of pH 7.6 **(A)**. The relative enzyme activity curve of TrUox was assessed under different pH buffers, including citric acid-NaH_2_PO_4_ buffers (pH 4.0–8.0) and Gly-NaOH buffers (pH 8.0–10.0), at a constant temperature of 35°C **(B)**. The relative enzyme activity curve of TrUox incubated at 4, 35, 37, and 50°C **(C)**. The relative enzyme activity curve of TrUox after incubation with pH 4.0~10.0 buffer for 12 and 24 h **(D)**.

TrUox was subjected to temperature gradient testing by incubating the enzyme with its substrate across a temperature gradient ranging from 20 to 65°C, in 5°C increments. The enzyme retained over 50% of its activity between 30 and 50°C, with optimal activity observed at 35°C where it maintained ~98% of its activity at 37°C (Figure 2A). For pH sensitivity, the enzyme was tested in buffers ranging from pH 3 to pH 10, with 0.6 pH unit intervals. TrUox showed consistent over 50% activity within a pH range of 6.0–9.0 (Figure 2B).

The thermostability of TrUox was evaluated by incubation at 4, 35, 37, and 50°C. The results demonstrated significant temperature- and time-dependent characteristics: When stored at 4°C, TrUox retained >90% residual activity for up to 18 days. At 35 and 37°C, the enzyme maintained >90% residual activity during the first 10 days of incubation. However, prolonged exposure to these elevated temperatures led to gradual structural degradation, resulting in a decline to 50% residual activity by day 18. Under the more severe thermal stress of 50°C, TrUox initially preserved >90% activity until day 4, but subsequently underwent irreversible denaturation, with residual activity plummeting to 50% by day 6 (Figure 2C).

Long-term pH stability was examined by incubating the enzyme at various pH levels (6.0 to 10.0) for 12 and 24 h, with no significant difference observed between the two time points, indicating consistent activity across this pH range. TrUox activity was significantly reduced at pH values below 4.0 or above 9.6 (Figure 2D). These results highlight the enzyme's adaptability to a range of environmental conditions.

**Figure 3:**
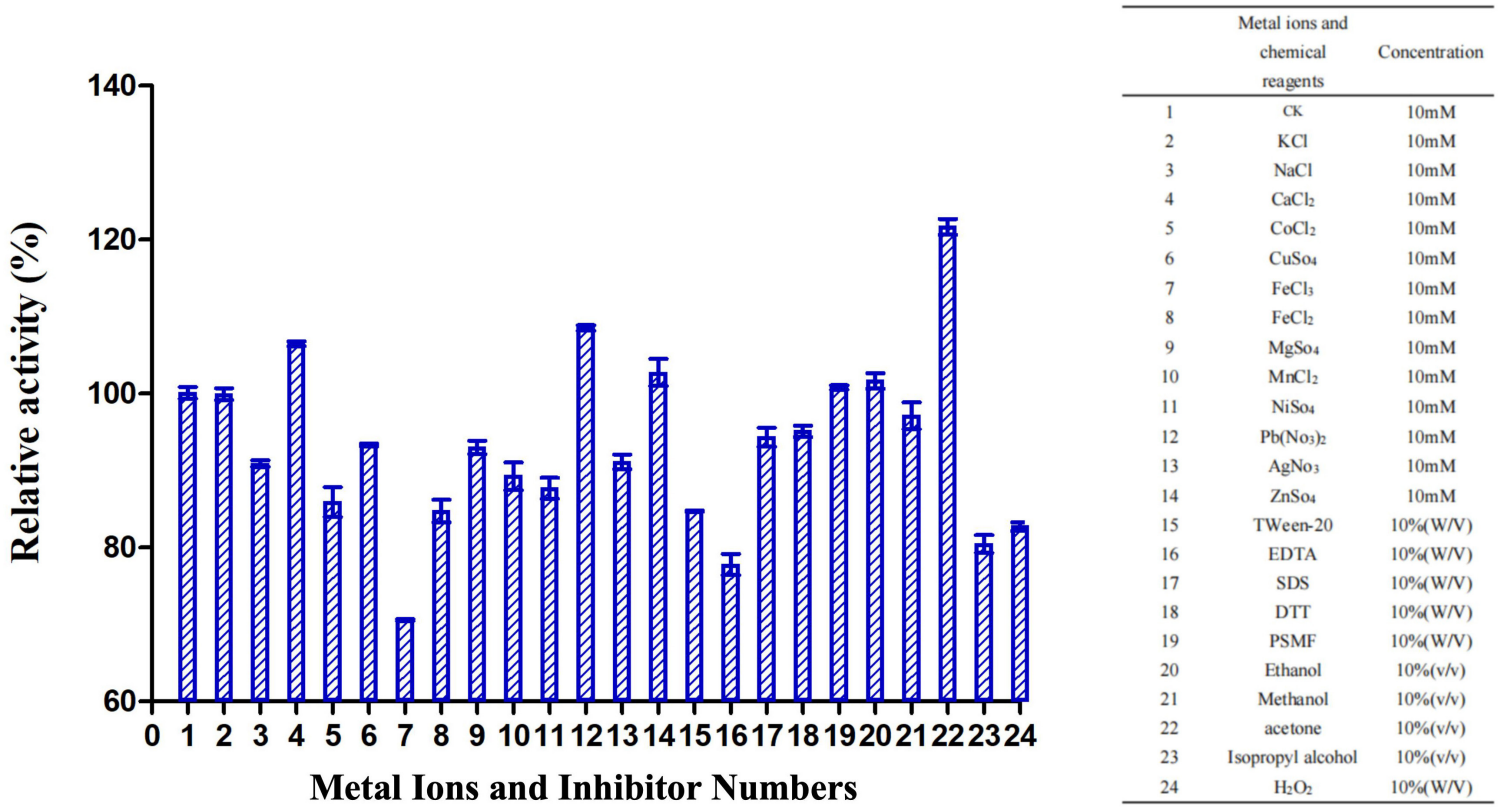
Effects of metal ions and inhibitors on TrUox activity. CK represents the sample without metal ions or inhibitors. The red ** indicates significant (*p* < 0.01) promotion, and the green ** indicates significant inhibition.

TrUox enzyme activity increased in the presence of metal ions Ca^2+^, Pb^2+^, and Zn^2+^, with relative activity exceeding 100%. Methanol exhibited the most significant stimulatory effect, increasing relative activity to over 120%. Conversely, chemicals such as PMSF, ethanol, and methanol showed no substantial impact on TrUox activity, while EDTA had an inhibitory effect, reducing relative activity to below 80% (Figure 3).

### Enzyme activity simulation in human physiological state

**Figure 4:**
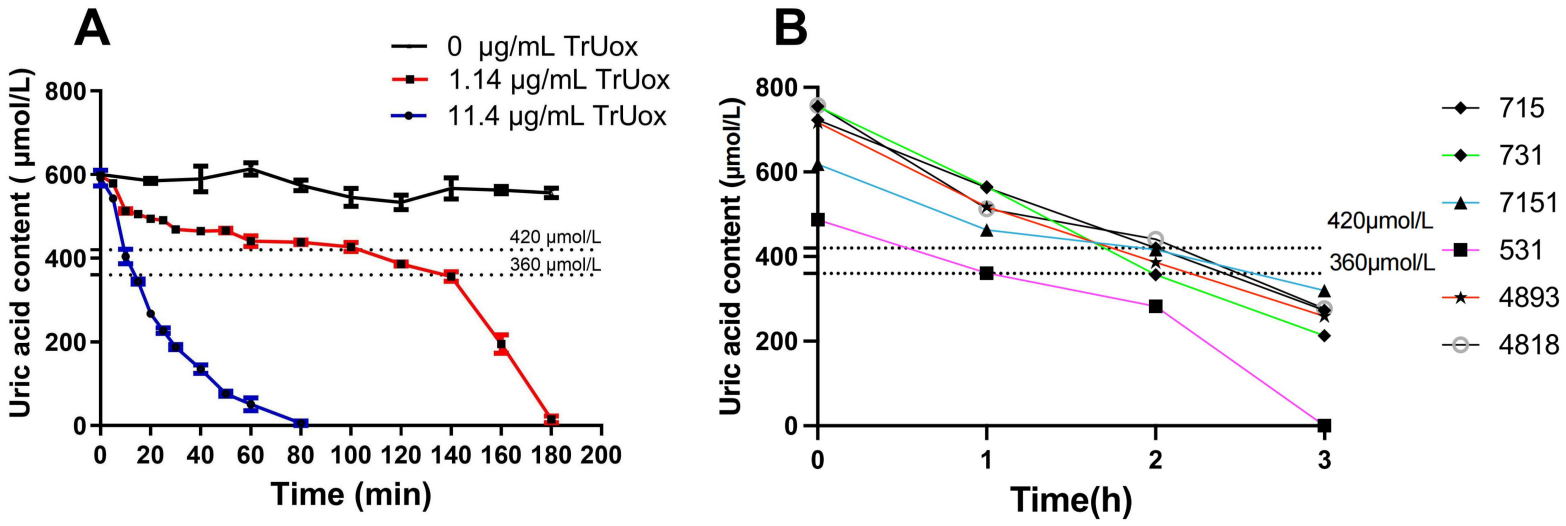
The effect of recombinant uricase TrUox on uric acid levels in normal saline and patient blood samples. **(A)** Degradation curves of uric acid at 37°C with TrUox concentrations of 0, 1.14, and 11.4 μg/mL in saline (0.9% NaCl). **(B)** Uric acid levels in patient blood samples with initial concentrations of 715, 731, 618, 487, 717, and 757 μM after TrUox addition to a final concentration of 1.14 μg/mL.

When blood levels of uric acid exceed the body's solubility threshold, urate crystals may precipitate and deposit in joints and periarticular cartilage, leading to specific diseases (Singh and Gaffo, 2020; Hansildaar et al., 2021). To replicate this physiological state *in vitro*, we introduced 11.4 μg/mL of TrUox into a high uric acid environment, using 600 μmol/L uric acid as the substrate. This treatment reduced the uric acid concentration to a normal range within 15 min. Additionally, using a lower concentration of 1.14 μg/mL TrUox, the uric acid level reached the normal range after 120 min and dropped to 0 μmol/L after 180 min (Figure 4A). These findings highlight the significant potential of TrUox for use in developing uric acid diagnostic kits and treating uric acid metabolic diseases.

Based on the degradation experiment of recombinant uricase TrUox in saline solution, this study selected anticoagulated whole blood samples from six hyperuricemic patients. For blood sampling, antecubital venous blood was collected from fasting subjects using heparin anticoagulant tubes. Immediately after collection, the tubes were gently inverted to ensure mixing and temporarily stored at 4°C. Uricase was subsequently added to the processed samples, achieving a final enzyme concentration of 1.14 μg/mL. All six patients exhibited blood uric acid levels below the reference range 2 h post-treatment (Figure 4B). This indicates that recombinant uricase TrUox can effectively reduce blood uric acid concentration in a short time.

### Homology modeling and quality evaluation of the predicted models

**Figure 5:**
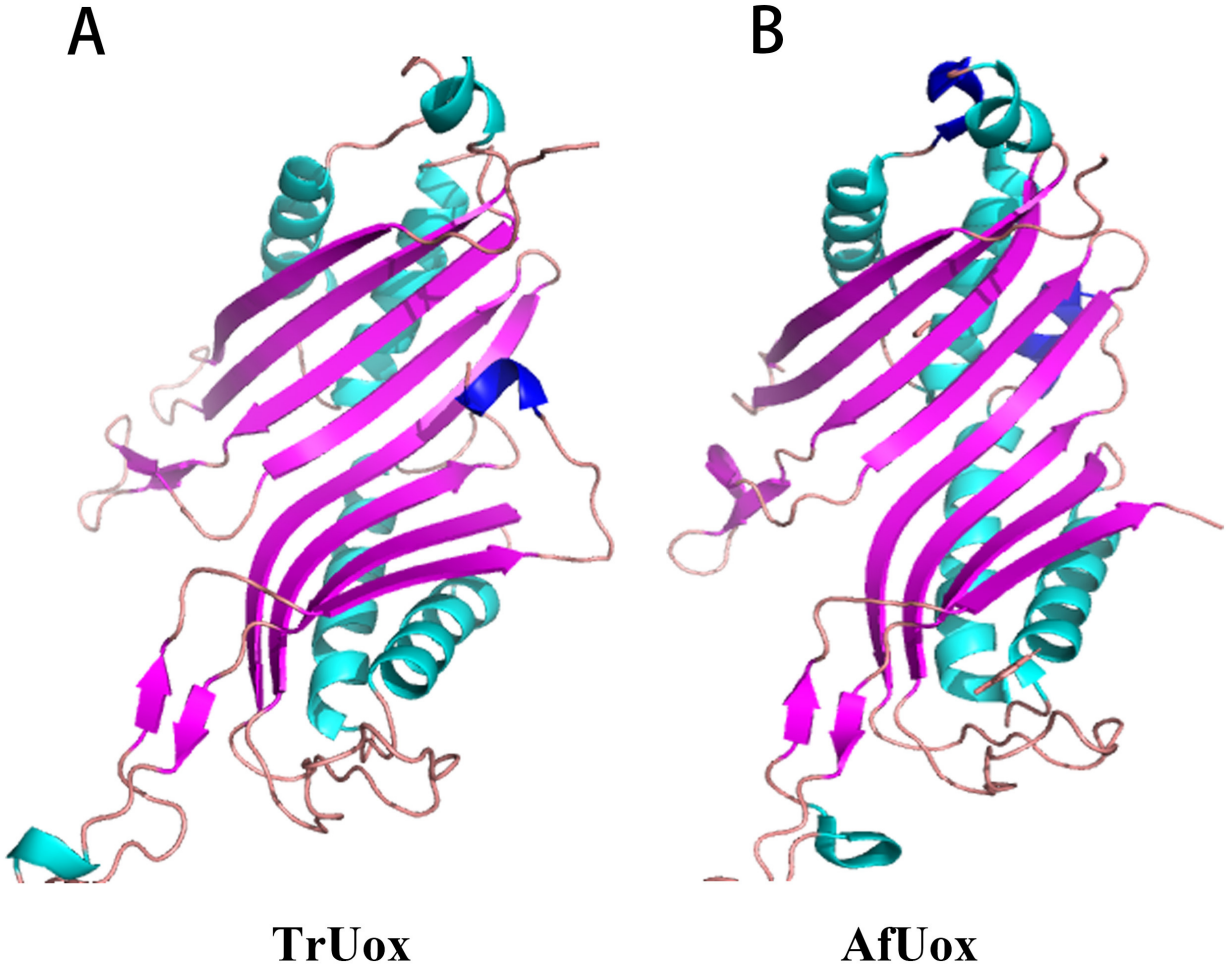
Monomer structure of TrUox **(A)** and AfUox **(B)**, with β-folded in purple and α helix in blue-green and dark blue.

The predicted protein model for TrUox was assessed with a QMEAN Z-score of −2.16, indicating a structure of good quality. Further evaluation using VERIFY 3D revealed that over 85.54% of the residues achieved a 3D-1D score of at least 0.2, confirming most residues are favorably positioned. ProQ analysis supported this with a ProQ LG score of 4.25, suggesting good stereochemistry of the TrUox structure. Ramachandran Plot, generated by PROCHECK, showed 96.2% of amino acid residues in the most favorable regions, underscoring the model's accuracy (Supplementary Figure S2). The overall quality factor as predicted by the ERRAT server was 95.66. These evaluations provide a solid foundation for subsequent structural studies. The 3D structure of TrUox was visualized using PyMOL (Figure 5A).

Sequence comparison results show that TrUox and AfUox share 34.2% amino acid sequence similarity, as depicted in Supplementary Figure S3. Both enzymes exhibit similar monomer structures, which include an eight-stranded β-sheet in sequence, complemented by two shorter β-strands, four primary α-helices, and two single-turn α-helices. Notably, TrUox features an additional half-turn α-helix toward the end of its amino acid sequence. In contrast, AfUox contains additional single-turn α-helical regions at positions 50 and 193, highlighted in dark blue on the structures (Figure 5).

### Flexibility analysis

**Figure 6:**
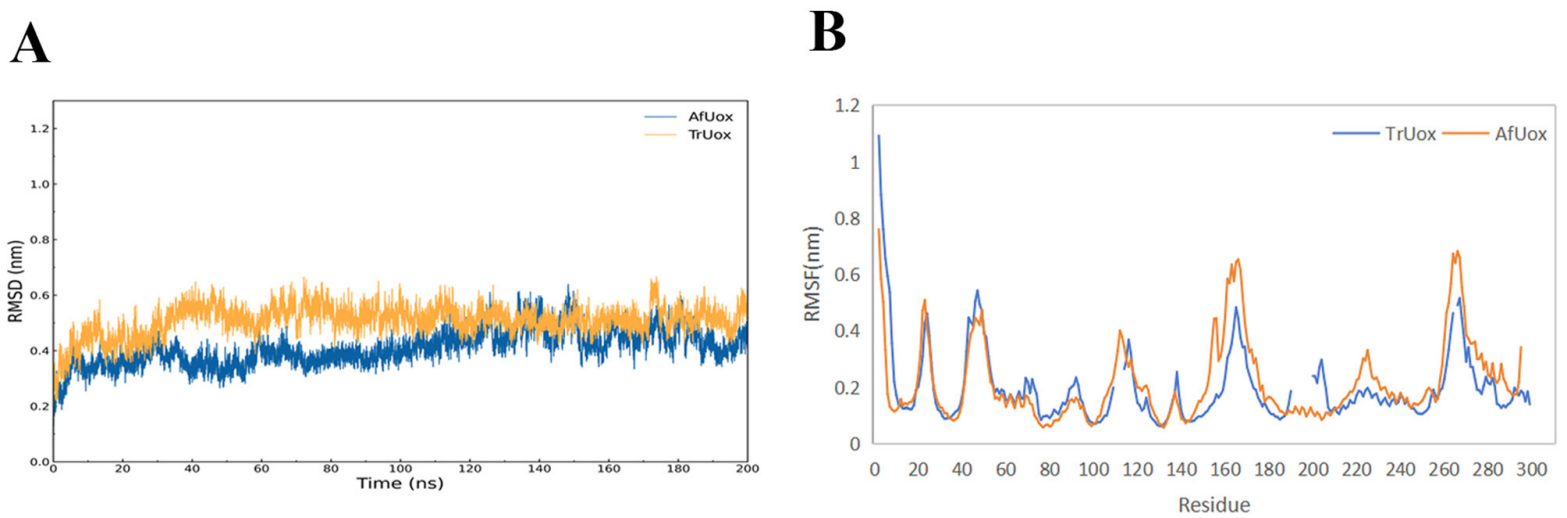
TrUox and AfUox as a function of time in MD simulations RMSD profiles **(A)**, and Per-residue average backbone RMSF profiles of TrUox and AfUox calculated from MD trajectories **(B)**.

The temperature chosen for the MD simulations of TrUox and AfUox was 310 K, which is the temperature at which uricase exerts its biological function. Using the initial structure of simulation as a reference, the RMSD values of the 200 ns complete trajectories were calculated. In the simulation process, not only was the overall RMSD value of TrUox was higher than that of AfUox during the simulation but also its curves showed less fluctuation (Figure 6A), which indicated that TrUox underwent fewer conformational changes. To further examine the molecular flexibility of these two enzymes, we calculated the RMSF values of their Cα atoms. As indicated in Figure 6B, the RMSF values of TrUox were lower than those of AfUox in most regions, and the average RMSF values of TrUox and AfUox were 0.195 and 0.216 Å, respectively, suggesting that TrUox showed higher structural rigidity and stability than AfUox during the 310 K simulations.

### Properties of the free energy landscape

**Figure 7:**
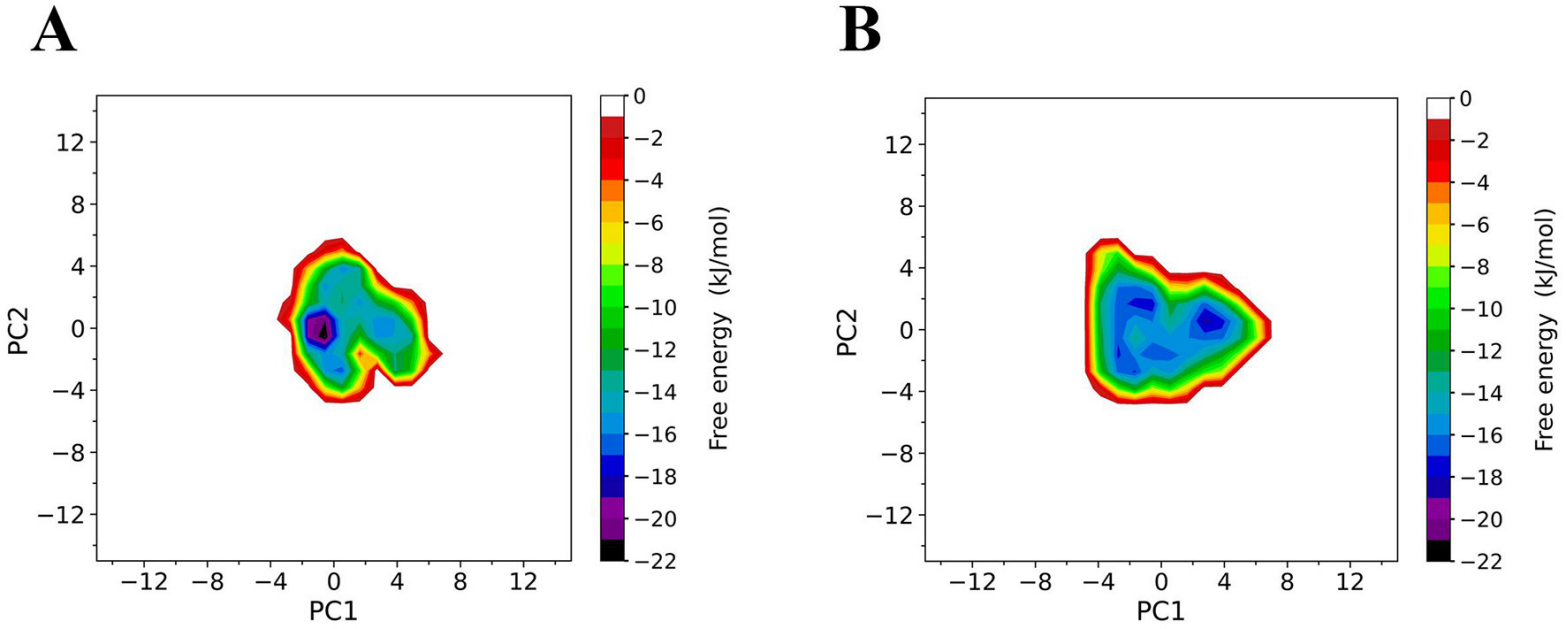
Constructed free-energy landscapes (FELs) of TrUox and AfUox. 2D FELs for TrUox **(A)** and AfUox **(B)** as a function of the first (PC1) and second (PC2) eigenvectors, respectively. The color bar represents the free-energy value in kJmol^−1^.

To compare the conformational distributions of TrUox and AfUox structures during simulations, two-dimensional free energy landscapes (FELs) were constructed (Figure 7). TrUox exhibits a smaller surface area of free energy and a lower global minimum free energy value, indicating reduced conformational entropy and diversity compared to AfUox. The global minimum of TrUox is relatively large and concentrated in the free energy graph, in contrast to AfUox, which has more free energy wells and dispersed global minima. These results indicated that TrUox has higher structural stability, which correlates with its enhanced thermal tolerance.

## Discussion

In our quest for a more heat-stable urate oxidase, we explored the complete genome of *T. rubra* YIM 77501^T^. Previous studies confirmed that this strain possesses cellulase and xylanase activities, with proteins expressed homogeneously exhibiting optimal reaction temperatures between 30 and 60°C and demonstrating stable heat resistance (Yin et al., 2017a,b). In addition, we used the pSHY211 constitutive expression vector, which avoids the toxic effects produced during IPTG induction and facilitates expression in the intestine.

The optimal temperatures for most of the studied microbial-derived uricases were in the range of 30–50°C (Table 1). The applications of uricases are mainly focused on enzymes for pharmaceutical use and uric acid detection kits, the study of thermostable uricases that can maintain high activity at medium temperatures has become a major research trend. The optimal temperature of TrUox is similar to that of uricase from *Bacillus subtilis* and *Candida utilis*, which is close to that of the human body. The activity of most microbial uricases is highly sensitive to the reaction pH, and the activity at pH 7.4 is usually < 50% of that at the optimal pH activity (Huang et al., 2015). TrUox maintains 90% relative activity at pH 7.4, with an optimal pH of 7.6. In temperature tolerance experiment, TrUox has the ability of long-term cryopreservation capabilities, and can also maintain the highest activity for a certain fermentation time for up to 4 days at the industrial fermentation temperature of 50°C, and the half-life can reach more than 18 days at 37°C (Figure 2C). Under optimal reaction conditions, TrUox achieved 32.29 ± 0.51 IU/mg enzyme activity, with a V_max_ of 59.61 ± 2.75 μmol.min^−1^.mg^−1^, *K*_m_ of 0.03 ± 0.00 μM, and K_cat_ of 33.73 ± 1.56S^−1^. The fitted straight line was calculated as y = 0.0005x + 0.0168 (Supplementary Figure S4). Compared with urate oxidase derived from *Candida utilis*, TrUox exhibits both a higher specific activity (32.29 ± 0.51 IU/mg) and a significantly reduced Michaelis constant Km (Bomalaski et al., 2002). These synergistic kinetic characteristics collectively demonstrate that TrUox achieves enhanced substrate-binding affinity. Compared to other microbial-derived urate oxidases listed in Table 1, TrUox demonstrates superior temperature tolerance. Combined with its enhanced thermostability, this enzyme exhibits significant potential for applications in enzyme preparation transport, long-term storage, and clinical therapeutic scenarios requiring high enzyme activity.

**Table 1:** Uricases from various microorganisms.

**Organism**	**Mw (kDa)**	**Opt. Tem**.	**Opt. pH**	**Stability**	***K_*m*_* (μM)**	***k_*cat*_* (s-1)**	**References**
*T. rubra* YIM 77501^T^	32	35°C	7.6	37°C, 10 d, 100%	0.52	0.019	This study
*A. adeninivorans* NBRC 10858	35	40°C	10	55°C, 30 min, 80%	29.15	151.16	Zhang et al., 2019
*B. subtilis* LMD 69.3	60	37°C	8	37°C, 12 h, 50%	ND	ND	Pfrimer et al., 2010
*C. utilis* strain CGMCC2.120	34.16	37°C	8.5	37°C, 24 h, 40%	33.7	ND	Liu et al., 2011
*D. Radiodurans* R1	33	30°C	9	55°C, 1 h, 10%	332.58 ± 57.94	17.49 ± 1.49	Chiu et al., 2021a
*K. marxianus*	< 35	42°C	9	40°C, 90 h, 79%	67.6	32.74	Wang et al., 2018
*B. firmus* DWD-33	33.5	50°C	8	60°C, 1 h, 100%	78.9	21.8x10^3^	Kotb, 2015
*Microbacterium* sp. strain ZZJ4-1	34	30°C	8.5	65°C, 0.5 h, 100%	310	3.01	Kai et al., 2008

Mw, molecular weight; Opt. Tem., Optimal temperature; Opt. pH, Optimal pH; ND, no mention.

As shown in Figure 4, the recombinant uricase TrUox can effectively reduce high uric acid levels in saline or blood to normal levels under physiological conditions. Currently, widely used uric acid-lowering drugs, such as allopurinol, colchicine, and febuxostat, may have side effects on liver and kidney function or pose potential risks to patients with coronary heart disease (Casanova et al., 2024). Therefore, in recent years, some researchers have developed recombinant uricase, such as rasburicase and pegloticase, as alternative drugs for the treatment of hyperuricemia and gout (Goldman, 2003; Garay et al., 2012; Naomi et al., 2023). The results of this study suggest that exogenous uricase supplementation can effectively regulate blood uric acid levels in both short-term and long-term treatments, providing new experimental evidence for the treatment of hyperuricemia. In addition, considering that the optimal reaction temperature and pH value of TrUox are close to human physiological conditions, the findings of this study provide strong theoretical support for its potential as a therapeutic drug for hyperuricemia. However, it should be noted that the current study is solely based on *ex vivo* experiments using human blood samples. The clinical translational potential still requires systematic validation through animal studies and clinical trials.

Uricase predominantly exists as a tetramer, with its four active sites located at the dimer interface. The thermal inactivation of this enzyme is primarily due to tetramer depolymerization and partial loss of secondary structure, where tetramer depolymerization plays a crucial role as the rate-limiting factor (Wu, 2016). It has been observed that the additional ring region present at the C-terminal of urate oxidase can interact spatially with the residues in its vicinity, thereby strengthening the inter-polymer interactions and enhancing thermostability (Pires et al., 2011; Feng et al., 2015). Both TrUox and AfUox exhibit similar secondary structures, however, TrUox features an additional ring region at the C-terminus (Figure 5A, dark blue region), similar to AgUox from *Arthrobacter globiformis* and TbUox from *Thermobispora bispora* (Shi et al., 2019; Chiu et al., 2021b). This additional ring region could be a contributing factor to its heat resistance.

Urate oxidase has a variety of conformational intermediates between dimers, which are linked through intramolecular non-covalent interactions, and then involve a large number of covalent bond breaks and conformational changes in the process of depolymerization (Wu, 2016). RMSD comparisons showed that TrUox exhibited smaller fluctuations than AfUox, indicating greater structural stability and reduced conformational changes (Sang et al., 2020). The lower root mean square fluctuation (RMSF) values of TrUox compared to AfUox further validate its enhanced structural stability, while its higher enzymatic activity (26.98 IU/mg) surpasses that of AfUox (Li et al., 2006). Thermophilic proteins generally tend to be less flexible than mesophilic proteins. However, Singh et al. (2015) proposed that thermophilic proteins balance rigidity and flexibility in order to maintain both stability and activity. The flexibility of its active site region can maintain or even improve catalytic activity, while the rigidity of other regions may help enhance thermostability. TrUox lower flexibility across most amino acid residues compared to AfUox aligns with its enhanced stability, as confirmed by RMSF analysis.

The free energy landscape (FEL) theory of protein-solvent systems provides the basis for in-depth understanding of protein dynamics and their interactions with solvents. FEL defines the thermodynamic properties (entropy and enthalpy changes) and kinetic properties (i.e., the free energy barrier between conformational states) of the protein-solvent system (Grottesi et al., 2002). FEL analysis for TrUox indicated a smaller free energy surface area, deeper funnel depth, and a more continuous free energy basin compared to AfUox, suggesting that TrUox has significantly lower conformational entropy and freedom, fewer conformational substates, and higher structural stability, which is consistent with the characteristics of other thermophilic enzymes.

It is noteworthy in subsequent fermentation production that TrUox was significantly inhibited by Fe^3+^ compared to other ions (relative activity < 50%), consistent with the *Bacillus* sp. and *Candida* sp. sourced uricase regarding inhibition of Fe^3+^ (Liu et al., 2011). The mechanism of action of Cu^2+^ on uricase is complex, some microbial sources of uricase have Cu^2+^-binding sites and mutations in His residues in the binding site (H-X-H-X-F) lead to loss of enzyme activity, but TrUox is less sensitive to 1 mmol/L Cu^2+^. In the same way as TrUox, some of the *Bacillus subtilis* and yeast-derived uricases have been previously studied to maintain their activity in the presence of Cu^2+^ (Wang et al., 2018).

## Conclusion

This study marks the first successful isolation and cloning of a functional urate oxidase gene (TrUox) from T*hermoactinospora rubra* YIM 77501^T^. The recombinant enzyme exhibits favorable thermostability, achieving optimal activity at 35°C and pH 7.6. Moreover, it demonstrates rapid uric acid degradation capability under *in vitro* whole-blood conditions. Structural analyses reveal that TrUox possesses enhanced rigidity, stability, and reduced conformational flexibility. These characteristics position it as a promising therapeutic candidate for the treatment of hyperuricemia. The findings of this research lay a solid foundation for the further advancement of TrUox in the fields of pharmaceutical development and enzyme-based therapies.

## Data Availability

The datasets presented in this study can be found in online repositories. The names of the repository/repositories and accession number(s) can be found in the article/Supplementary material.
