# Supplementary material for: Characterization of a thermostable uricase derived from Thermoactinospora rubra YIM 77501T and its heat-resistant mechanism
Source: Front Microbiol. 2025 Jun 19;16:1615845. doi: 10.3389/fmicb.2025.1615845 (PMC12225646; doi:10.3389/fmicb.2025.1615845)
Supplement: Supplementary file 1 [file Data_Sheet_1.pdf]

Characterization of a Thermostable Uricase Derived from *Thermoactinospora rubra* YIM 77501^{T} and Its Heat-resistant Mechanism

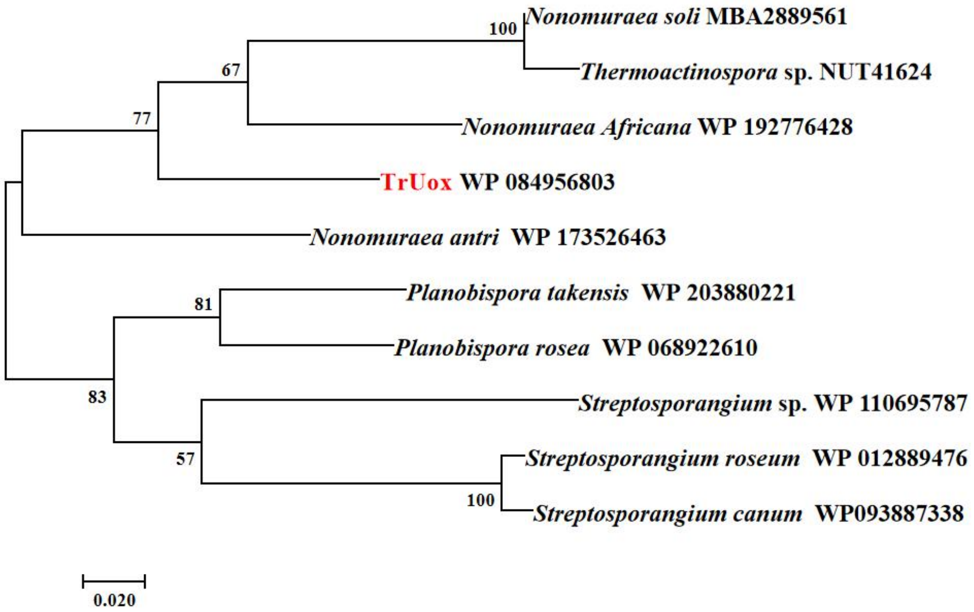

Supplementary Figure S1 Phylogenetic relationship between TrUox and related uricases. Bootstrap values (expressed as a percentage of 1000 replicates) are given at the nodes.

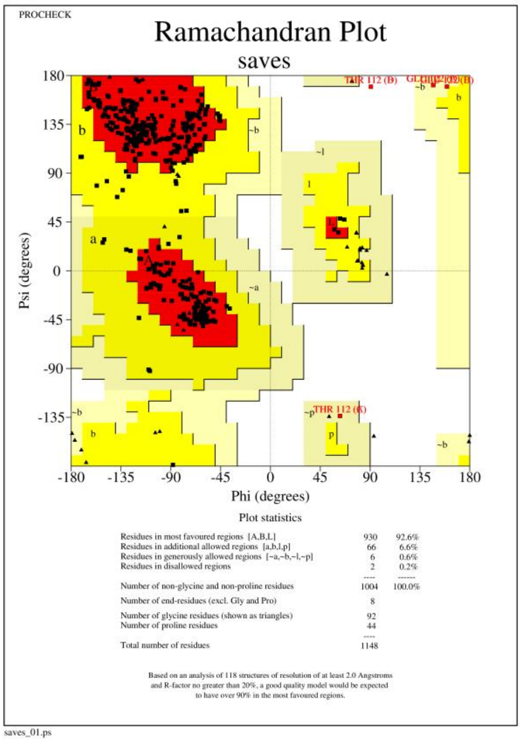

Supplementary Figure S2 Ramachandran Plot of TrUox

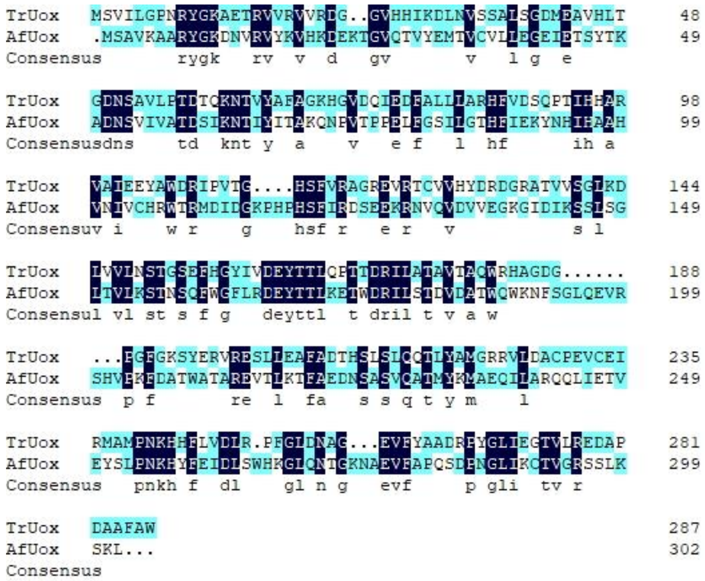

Supplementary Figure S3 Amino acid sequence comparison of TrUox and AfUox

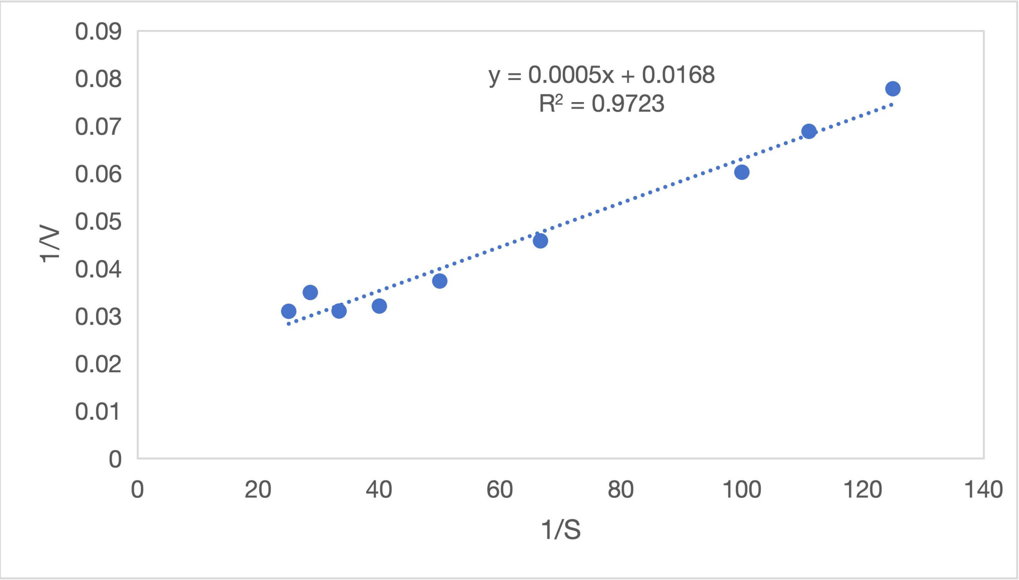

Supplementary Figure S4 Lineweaver-Burk plot of TrUox
