# Supplementary material for: Characterization of a thermostable uricase derived from Thermoactinospora rubra YIM 77501T and its heat-resistant mechanism
Source: Front Microbiol. 2025 Jun 19;16:1615845. doi: 10.3389/fmicb.2025.1615845 (PMC12225646; doi:10.3389/fmicb.2025.1615845)
Supplement: Supplementary file 2 [file Data_Sheet_2.pdf]

医学伦理审查批准书

Affidavit of Approval of Medical Ethics and Welfare

批准编号 Approval No.	MECDU-202410-27

本《实验方案》经过大理大学医学伦理委员会审核，符合医学实验伦理原则，符合国家医学实验伦理福利的相关规定。实验方案的相关信息如下：

This Experimental Protocol has been reviewed by the Medical Ethics Committee of Dali University and conforms to the principles of medical experiment ethics and the relevant provisions of the national medical experiment ethics and welfare. The relevant information of the experimental program is as follow:

课题名称及编号<br>Protocol Title and Number	云南热泉嗜热放线菌多样性及其碳代谢机制研究（编号. 230212528080）<br>Diversity and carbon metabolism mechanism of thermophilic actinomycetes in Yunnan hot spring（NO.230212528080）				
申请人<br>Applicant	尹以瑞<br>Yin Yirui	职称/学位<br>Title/Degree	副教授/博士<br>Associate Professor/Doctor	邮箱<br>Email	yinyirui@dali.edu.cn
课题负责人<br>Principle Investigator (PI)	尹以瑞<br>Yin Yirui	职称/学位<br>Title/Degree	副教授/博士<br>Associate Professor/Doctor	邮箱<br>Email	yinyirui@dali.edu.cn
院系(部门)<br>Institution	大理大学农学与生物科学学院<br>College of Agriculture and Biological Science, Dali University			申请日期<br>Application date	2024年10月24日<br>October 24, 2024
实验主要内容<br>experimental design	从环境微生物中获得尿酸氧化酶（UOX）、超氧化酶歧化酶（SOD）等功能酶，并将其在体外添加到人体来源抗凝血中，检测功能酶对血液中尿酸等指标的影响。<br>Functional enzymes such as urate oxidase (UOX) and superoxidase dismutase (SOD) were obtained from environmental microorganisms and added to human anticoagulant in vitro to detect the effects of functional enzymes on blood uric acid and other indexes.				
计划执行时间<br>Period of Protocol	2024/12-2027/05				
审查意见<br>Results of inspection	☑符合医学伦理福利要求，可以进行实验 Approval<br>☐调整方案后，可以进行实验 Resubmit after modification<br>☐不符合医学伦理福利要求，不可以进行实验 Rejection				
备注<br>Remarks	☑ 初审 ☐ Initial Review<br>☐ 第 次 审查 ☐ Review Cycle: Number:				

大理大学医学伦理委员会

Medical Ethics Committee of Dali University (Seal)

日期 Date 2024.10.27

地址：中国，云南省大理市，古城弘圣路 2 号，大理大学，邮编：671003
Add: NO.2 Hongsheng Road, Dali, Yunan 671003 P.R. CHINA, 671003
